# Sulfonylated Goniothalamin and Piplartine Derivatives Exhibit Selective Antiproliferative Activity on Breast Cancer Cells via Oxidative Stress-Mediated Mechanisms

**DOI:** 10.1021/acsomega.6c00212

**Published:** 2026-07-07

**Authors:** Julia Louise Moreira Nacif, Aloisio de Andrade Bartolomeu, Simone da Silva Lamartine-Hanemann, Bruno Zavan, Luiza de Mello Nascimento, Ester Siqueira Caixeta, Alexandre Ferro Aissa, Ronaldo Aloise Pilli, Marisa Ionta

**Affiliations:** † Laboratório de Avaliação de Protótipos Antitumorais (LAPAN), Instituto de Ciências Biomédicas, 74347 Universidade Federal de Alfenas, Alfenas, Minas Gerais zip code 37-130-001, Brazil; ‡ Instituto de Química, 28132 Universidade Estadual de Campinas (UNICAMP), Campinas, SP zip code 13083-970, Brazil

## Abstract

Breast cancer is a highly heterogeneous and treatment-resistant malignancy, underscoring the urgent need for novel therapeutic strategies. In this study, we investigated the antitumor potential of sulfonylated derivatives of goniothalamin (compound **3**) and piplartine (compound **6**) using the MCF-7 human breast cancer cell line as a study model. Both compounds significantly reduced cell viability of MCF-7 cells, with half-maximal inhibitory concentrations (IC_50_) of 20 μM (compound **3**) and 7 μM (compound **6**). These compounds were highly selective toward tumor cells (MCF-7) compared with nontumor cells (primary fibroblasts). Investigation of the mechanism of action revealed that both compounds exhibit pro-oxidant activity on tumor cells, contributing to DNA damage and cell cycle arrest. Key cell-cycle regulators, such as cyclins B and D, p21, and MYC, were modulated by the treatments. Interestingly, compound **3** induced cellular senescence, whereas compound **6** primarily triggered apoptosis. The expression profile of pro-apoptotic BAX and antiapoptotic BCL2 genes was also modulated by compound **6**. These findings suggest that the antitumor effects of compounds **3** and **6** are mediated by oxidative stress-induced DNA damage, leading to activation of senescence or apoptotic pathways. These results support further in vivo studies to validate the antitumor potential of sulfonylated derivatives of GNT and PPT in breast cancer.

## Graphical abstract

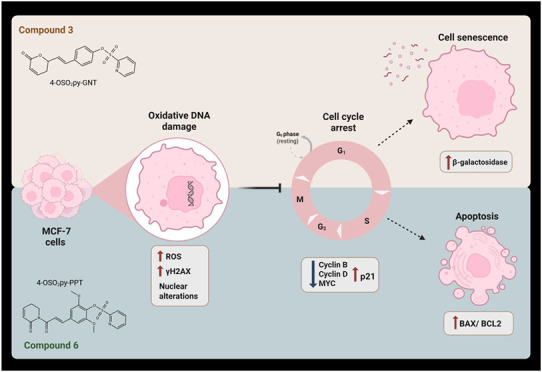

## Introduction

Breast cancer (BC) is the most frequently diagnosed malignancy and the leading cause of cancer-related death among women worldwide. In 2022, approximately 2.3 million women were diagnosed with the disease, and 670,000 deaths were reported globally.^1,2^ Distinct BC subtypes are primarily defined by the expression levels of hormone receptors (estrogen receptor α, ERα; and progesterone receptor, PR), as well as the human epidermal growth factor receptor 2 (HER-2). However, molecular profiling of BC tumors, based on omics analyses, has revealed a broader diversity of subtypes, such as luminal A, luminal B, HER2-enriched, basal-like, claudin-low, and normal-like.^3,4^ These molecular differences strongly influence patient prognosis, therapeutic response and disease progression.^5,6^

Luminal A tumors represent the most prevalent subtype of BC, accounting for approximately 60% of diagnosed cases. Although these tumors typically respond to endocrine therapy, therapeutic resistance frequently emerges through several mechanisms, including *ESR1* mutations, hyperactivation of the PI3K/AKT signaling pathway, and tumor plasticity.^7,8^ HER2-enriched and triple-negative subtypes are generally more aggressive and often require targeted or cytotoxic treatment strategies. However, treatment toxicity and tumor resistance remain significant clinical challenges across these breast cancer subtypes. Therefore, the development of therapeutic strategies that are both mechanistically innovative and more selective remains an important priority.^9,10^

Natural products represent a rich source of bioactive scaffolds for anticancer drug discovery, which may exhibit unique mechanisms of action that target specific cellular vulnerabilities.^11–13^ Among them, goniothalamin (GNT) and piplartine (PPT), also known as piperlongumine, have attracted attention due to their ability to modulate tumor cell proliferation and survival.^14,15^

GNT is a styryl lactone isolated from species of the *Goniothalamus* and *Cryptocarya* genera that exhibits cytotoxic activity across multiple cancer models, including breast, prostate, and colon cancers.^16–18^ The cytotoxicity of this compound has been associated with its ability to induce DNA damage, oxidative stress, and apoptosis.^19–21^ However, these effects are not selective toward cancer cells, which limits its therapeutic potential.^22,23^ PPT is an alkaloid/amide derived from *Piper* species that displays a broad spectrum of pharmacological activities, including anti-inflammatory, antinociceptive, and cytotoxic effects across several tumor models.^24–28^ Mechanistic studies have shown that PPT induces oxidative stress, autophagy, and apoptosis, while also inhibiting tumor cell migration and invasion.^29–31^ Despite these promising properties, limitations such as poor aqueous solubility and limited bioavailability remain important challenges for its clinical application.^32,33^

Accordingly, the rational chemical modification of natural compounds with promising pharmacological properties has emerged as an important strategy to overcome limitations associated with their clinical application.^34–36^ We have previously demonstrated that derivatives of GNT and PPT (4-OH-GNT and 4-OH-PPT) containing an electron-donating hydroxyl group at C-4 of the aromatic ring did not exhibit significant improvements in tumor cell selectivity compared with their parent compounds.^37^ However, the hydroxyl group also provides a suitable site for further structural modification, particularly the introduction of electron-withdrawing substituents to modulate the electronic properties.

The sulfonyl group is a strongly robust substituent and it has been widely explored in medicinal chemistry due to its ability to modulate physicochemical and electronic properties.^38,39^ In particular, the 2-sulfonyl pyridyl combines inductive effect (-I), a strong resonance effect (-M), decreased nitrogen basicity, high dipole moment and low LUMO energy which are associated with increased electrophilicity. The combined effect of the sulfonyl and pyridyl moieties influences key physicochemical properties, while enhancing potential interactions with biological targets.^40–42^ The 4-sulfonyl pyridyl group also acts as an electron-withdrawing group but the conjugation between the sulfonyl group and the pyridine ring is expected to be less effective, and its basicity is higher than its 2-sulfonyl analogue. On the other hand, the 3-sulfonyl pyridyl group shows a distinct electronic profile compared to the 2- and 4-sulfonyl analogues as the sulfonyl group is less effectively conjugated with the nitrogen lone pair of the pyridyl group, its electronic effect being transmitted mainly by inductive effect (-I). As a result, the nitrogen atom of the 3-sulfonyl pyridyl group is more basic than its 2- and 4-sulfonyl analogues, being protonated under mildly acidic conditions. Taking up all these considerations and the calculated values for some of the physicochemical parameters (see, Table S1, in the Supporting Information), we proposed the introduction of a 2-pyridyl sulfonyl substituent at the C-4 position of 4-OH-GNT (**2**) and 4-OH-PPT (**5**).

To enable this structural modification, we employed the sulfur­(VI) fluoride exchange (SuFEx) click chemistry approach, a robust and efficient method for the synthesis of sulfonylated derivatives.^43,44^

In this study, we designed and synthesized 2-sulfonyl pyridyl derivatives of goniothalamin and piplartine and investigated their antitumor potential using luminal A breast cancer cell line MCF-7 as study model.

## Materials and Methods

### Cell Lines and Culture Conditions

MCF-7 breast cancer cell line, representative of the luminal A subtype, was used. The primary dermal fibroblasts from healthy human skin (CCD-1059Sk) were used as the nontumorigenic control, and the experiments were performed between passages 6 and 12. Both cell lines were obtained from the Rio de Janeiro Cell Bank (Brazil). Cells were cultured in Dulbecco’s Modified Eagle’s Medium/Ham’s F12 (DMEM/F12; Sigma-Aldrich) supplemented with 10% fetal bovine serum (FBS; Cultilab). Cultures were maintained in a humidified incubator at 37 °C under 5% CO_2_. Cell stocks were preserved in liquid nitrogen until use.

### Synthesis of Sulfonyl Derivatives of Goniothalamin and Piplartine

rac-Goniothalamin (GNT) (**1**), rac-4-hydroxygoniothalamin (**2**), piplartine (**4**), and 4-hydroxypiplartine (**5**) were synthesized in our laboratory following previously reported procedures.^37,45^ For the synthesis of the sulfonylated derivatives **3** and **6**, the selection of the most suitable sulfonyl substituent was guided by a qualitative *in silico* evaluation considering drug-likeness parameters, including compliance with Lipinski’s Rule of Five (Ro5) and the Rule of Three (Ro3), as well as predicted activity, selectivity, and ligand efficiency metrics (Table S1). Figure 1 shows the chemical structures of the natural compounds goniothalamin (GNT, **1**) and piplartine (PPT, **4**), as well as their hydroxylated derivatives 4-OH-GNT (**2**) and 4-OH-PPT (**5**) and their sulfonylated derivatives 4-(2′-SO_2_pyr)­O-GNT (**3**) and 4-(2′-SO_2_pyr)­O–PPT (**6**).

**Figure 1:**
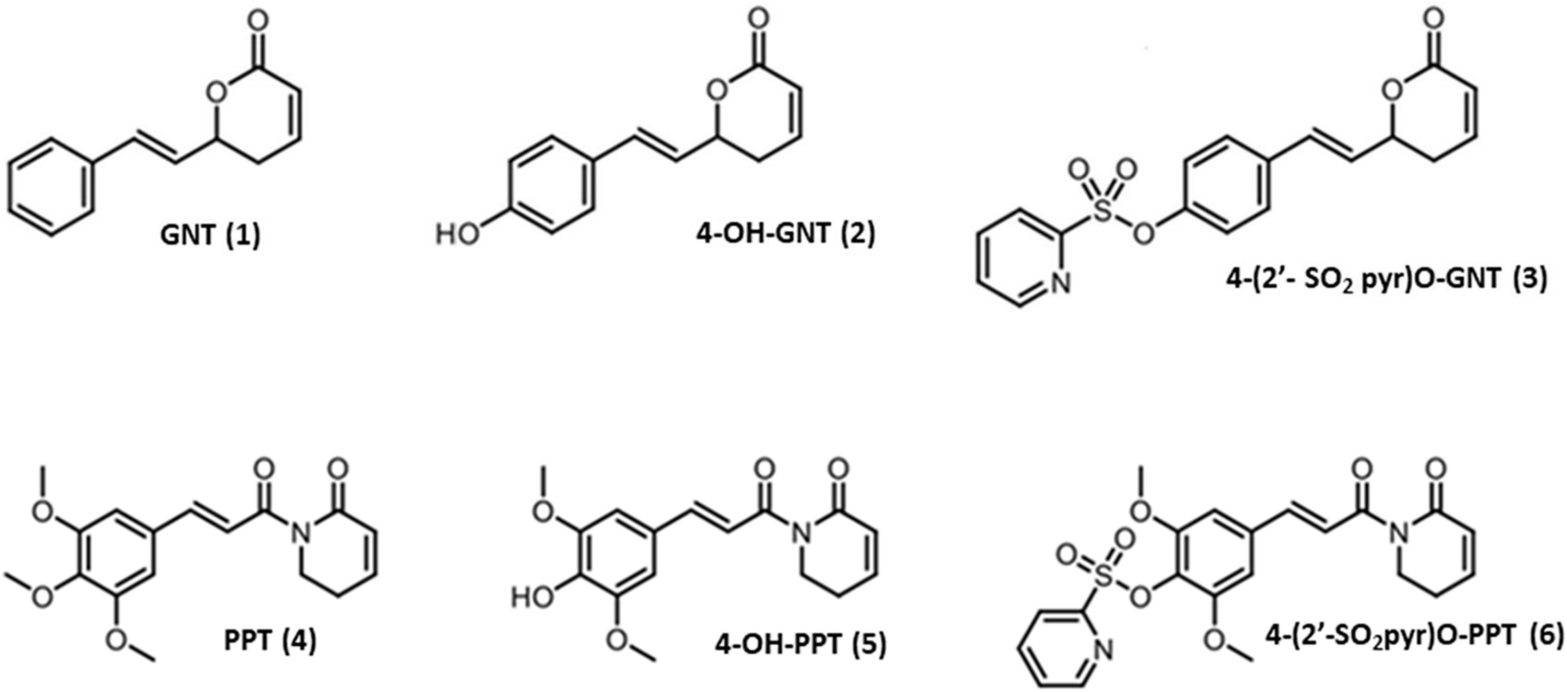
Chemical structures of the natural products goniothalamin (GNT, **1**) and piplartine (PPT, **4**); their hydroxylated derivatives 4-OH-GNT (**2**) and 4-OH-PPT (**5**); and their sulfonylated derivatives 4-(2′- SO_2_ pyr)­O–GNT (**3**) and 4-(2′-SO_2_pyr)­O–PPT (**6**).

Compounds **3** and **6** were synthesized via a sulfur­(VI) fluoride exchange (SuFEx) reaction between pyridine-2-sulfonyl fluoride (PyFluor) and the corresponding 4-hydroxy derivatives **2** and **5**, using Cs_2_CO_3_ as the base (Scheme 1). Typically, the phenolic substrate (0.1 mmol), PyFluor (19.3 mg, 0.12 mmol), Cs_2_CO_3_ (65.2 mg, 0.2 mmol), and dry MeCN (350 μL) were added to a screw-cap test tube (solid top with PTFE liner) equipped with a magnetic stirring bar. The tube was purged with nitrogen, sealed, and the reaction mixture stirred at room temperature for 1 h. After completion, the solvent was removed under reduced pressure, and the residue was purified by flash column chromatography on silica gel. For compound **3**, a gradient of 40–70% EtOAc in hexanes afforded a yellow oil (33.1 mg, 93%). For compound **6**, a gradient of 50–80% EtOAc in hexanes afforded a white solid (41.1 mg, 92%).

**Scheme 1:**
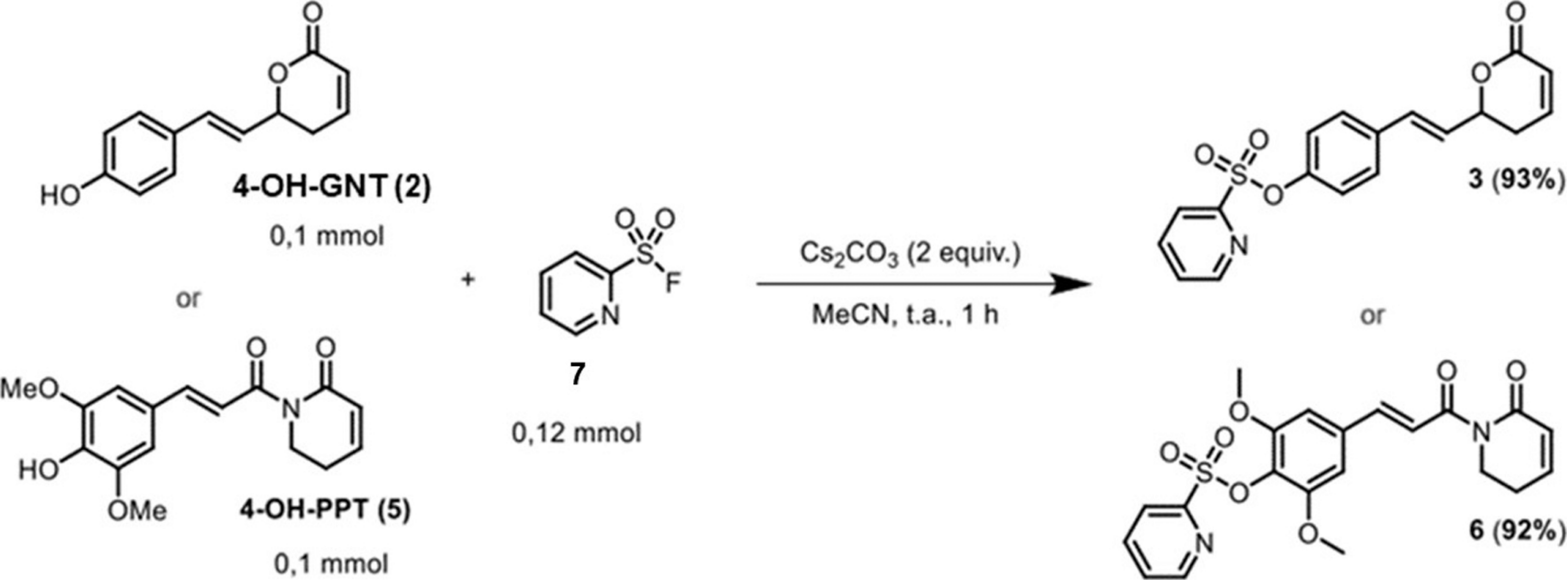
Synthetic Routes for Obtaining Sulfonylated Derivatives of GNT and PPT^a^

a Reaction of 4-OH-GNT (**2**) or 4-OH-PPT (**5**) with pyridine-2-sulfonyl fluoride (**7**) in the presence of Cs_2_CO_3_ afforded the corresponding GNT and PPT derivatives **3** and **6**, respectively, in 93% and 92% yields

#### *rac*-(*E*)-4-(2-(6-oxo-3,6-Dihydro-2*H*-Pyran-2-yl)­Vinyl)­Phenyl Pyridine-2-Sulfonate (**3**)

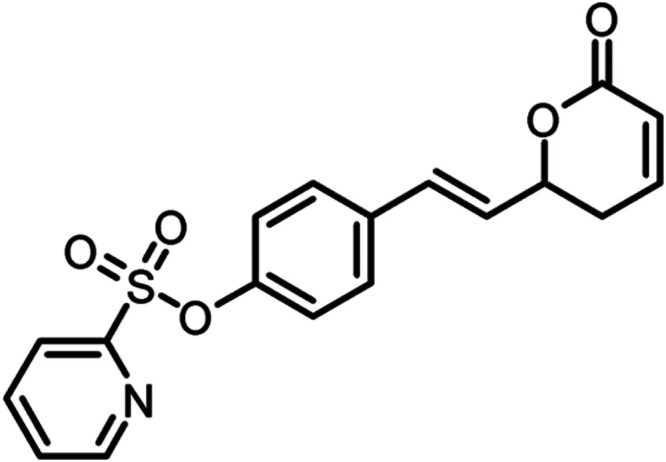



FTIR (thin film): *ṽ* = 1720, 1580, 1502, 1429, 1379, 1247, 1203, 1183, 1150, 1118, 1086, 1059, 1017, 971, 870, 841, 818, 761, 736, 698 cm^–1^; ^1^H NMR (250 MHz, CDCl_3_): δ = 8.82 (d, *J* = 4.7 Hz, 1H), 8.02–7.88 (m, 2H), 7.60 (ddd, *J* = 6.8, 4.7, 1.9 Hz, 1H), 7.32 (d, *J* = 8.7 Hz, 2H), 7.09 (d, *J* = 8.6 Hz, 2H), 6.91 (dt, *J* = 9.0, 4.2 Hz, 1H), 6.68 (d, *J* = 16.4 Hz, 1H), 6.20 (dd, *J* = 16.0, 6.1 Hz, 1H), 6.08 (dt, *J* = 9.8, 1.8 Hz, 1H), 5.15–5.01 (m, 1H), 2.61–2.44 (m, 2H) ppm; ^13^C NMR (63 MHz, CDCl_3_): δ = 163.8, 153.6, 150.7, 149.5, 144.8, 138.3, 135.1, 131.6, 128.2, 128.0, 127.0, 124.4, 122.7, 121.7, 77.7, 29.9 ppm; HRMS (FTMS + pESI) *m*/*z*: [M + H]^+^ Calcd. for C_18_H_16_NO_5_S^+^ 358.07437; Found 358.07446 (Figures S1–S3).

#### (*E*)-2,6-Dimethoxy-4-(3-Oxo-3-(6-Oxo-3,6-Dihydropyridin-1­(2*H*)-yl)­Prop-1-en-1-yl)­Phenyl Pyridine-2-Sulfonate (**6**)

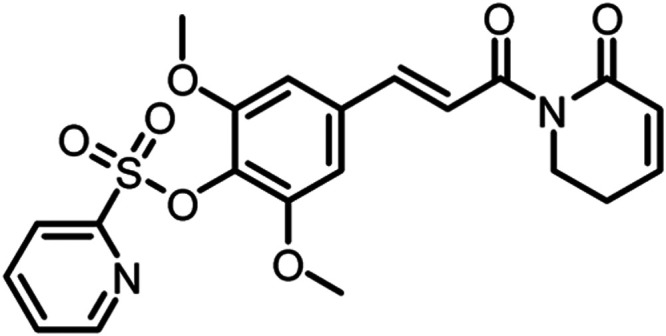



m.p.: 187–188 °C; FTIR (thin film): *ṽ* = 1685, 1619, 1594, 1499, 1464, 1426, 1382, 1323, 1282, 1249, 1218, 1185, 1131, 861, 825, 765, 736, 696 cm^–1^; ^1^H NMR (250 MHz, CDCl_3_): δ = 8.79 (d, *J* = 4.7 Hz, 1H), 8.07–7.90 (m, 2H), 7.67–7.53 (m, 2H), 7.40 (d, *J* = 15.5 Hz, 1H), 6.95 (dt, *J* = 9.8, 4.1 Hz, 1H), 6.71 (s, 2H), 6.02 (dt, *J* = 9.7, 1.9 Hz, 1H), 4.02 (t, *J* = 6.5 Hz, 2H), 3.64 (s, 6H), 2.54–2.41 (m, 2H) ppm; ^13^C NMR (63 MHz, CDCl_3_): δ = 168.7, 166.0, 155.6, 153.4, 150.0, 145.9, 142.7, 137.9, 134.8, 129.6, 127.5, 125.8, 123.5, 123.1, 105.0, 56.3, 41.8, 24.9 ppm; HRMS (FTMS + pESI) *m*/*z*: [M + H]^+^ Calcd. for C_21_H_21_N_2_O_7_S^+^ 445.10640; Found 445.10591 (Figures S4–S6).

### Cell Viability Assay

Cell viability was assessed using the sulforhodamine B (SRB) assay.^46^ MCF-7 and CCD-1059Sk cells (1 × 10^4^ cells/well, 96-well plates) were treated for 48 h with compounds **1**, **3**, **4**, and **6** (0.01–200 μM). Tamoxifen (0.01–150 μM) and doxorubicin (0.01–20 μM) were used as positive controls. Cells were fixed with 10% (w/v) trichloro acetic acid (1 *h*, 4 °C), stained with SRB (0.4% in 1% acetic acid, 1 h), washed with 1% (v/v) acetic acid, and dye was solubilized in 10 mM Tris-base (30 min). Absorbance was measured at 540 nm (reference of 690 nm). The half-maximal inhibitory concentration (IC_50_) values were calculated using GraphPad Prism 8.0 software (GraphPad Software, Inc.). The selectivity index (SI) was determined as IC_50_ (CCD-1059Sk)/IC_50_ (MCF-7).

Cell viability assays were also performed to investigate the role of oxidative stress. MCF-7 cells were treated with compound **3** (20 μM) or compound **6** (7 μM), with or without *N*-acetyl-l-cysteine (NAC, 1 mM; Sigma-Aldrich). A NAC-only control was included. After 48 h, viability was determined using the SRB as described above.

### Clonogenic Assay

The clonogenic assay was performed according to Franken et al. (2016).^47^ MCF-7 cells (300 cells/35 mm plate) were treated with compound **3** (10 and 20 μM) or **6** (3.5 and 7 μM) for 48 h, followed by an 11-day recovery in drug-free medium. Colonies were fixed with methanol (30 min), stained with 0.5% crystal violet (5 min), and counted under a stereomicroscope (Nikon SMZ800). Colonies with >50 cells were considered for analysis.

### Cell Cycle Analysis

MCF-7 cells (1 × 10^5^/35 mm plate) were treated with compound **3** (10 and 20 μM) or compound **6** (3.5 and 7 μM) for 48 h. Cells were harvested by trypsinization (Trypsin-EDTA, Sigma-Aldrich), fixed in 75% ethanol at 4 °C overnight, and stained with a solution containing RNase (1.5 mg/mL) and propidium iodide (90 μg/mL) for 1 h at 4 °C. DNA content was analyzed on a Guava EasyCyte 8HT flow cytometer (Hayward) using the manufacturer’s software.

### Immunofluorescence

MCF-7 cells (1 × 10^5^/35 mm plate) were seeded on coverslips and treated with compound **3** (10 and 20 μM) or compound **6** (3.5 and 7 μM) for 48 h. Cells were fixed in 3.7% formaldehyde (30 min) and permeabilized with 0.5% Triton X-100 in PBS (15 min). Samples were incubated overnight at 4 °C with primary antibodies: anti-α-tubulin (1:100, Cell Signaling Technology, #3873) or anti-γH2AX (1:100, CST, #9718). The next day, cells were incubated with secondary antibodies for 2 h at room temperature: antimouse IgG-FITC antibody (1:100, Sigma-Aldrich, F0257) or antirabbit IgG Alexa Fluor 488 (1:100, Abcam, #ab150073). Actin filaments were labeled with phalloidin-TRITC (Sigma-Aldrich) for 2 h and nuclei were counterstained with DAPI (Fluoroshield, Sigma-Aldrich, #F6057). Images were acquired using confocal or epifluorescence microscopy and analyzed with NIS-elements AR software (version 5.21.03, Nikon).

### Annexin V Assay

Apoptosis was assessed using the Alexa Fluor 488 Annexin V/Dead Cell Apoptosis Kit (Thermo Fisher Scientific) according to the manufacturer’s instructions. MCF-7 cells (1 × 10^5^ cells/35 mm plate) were treated with compound **3** (10 and 20 μM) or **6** (3.5 and 7 μM) for 48 h. Cells were harvested by trypsinization and incubated with Annexin V-AF488 and propidium iodide for 30 min at room temperature in the dark. Samples were analyzed by flow cytometry (Guava EasyCyte 8HT, Merck Millipore) using GuavaSoft 2.7 software.

### Cellular Senescence Assay

Cellular Senescence was evaluated using the Senescence β-Galactosidase Staining Kit (Cell Signaling Technology, #9860). MCF-7 cells (1 × 10^5^ cells/35 mm plate) were treated with compound **3** (10 and 20 μM), and cell morphology was monitored at 0, 24, 48, and 72 h. After 72 h, cells were fixed in 1 mL of the kit-provided fixative solution for 15 min, followed by incubation with 1 mL of β-galactosidase staining solution at 37 °C in a CO_2_-free environment for 7 h. Senescence-associated β-galactosidase activity was observed by phase-contrast microscopy, and images were acquired using a blue filter.

### CellROX Assay

Reactive oxygen species (ROS) levels were measured using the CellROX Green Flow Cytometry Assay Kit (Invitrogen, #C10444). MCF-7 cells (1 × 10^5^ cells/35 mm plate) were treated with compound **3** (20 μM) or **6** (7 μM) for 4 h. Hydrogen peroxide (1 mM, 30 min) was used as a positive control. Following treatment, cells were incubated with CellROX Green Reagent (5 μM, 30 min, 37 °C), harvested, and analyzed by flow cytometry (Guava EasyCyte 8HT, Merck Millipore) using GuavaSoft 2.7.

### RT-qPCR

MCF-7 cells (2 × 10^5^ cells/35 mm plate) were treated with compounds **3** (10 and 20 μM) or **6** (3.5 and 7 μM) for 24 h. Total RNA was extracted using the RNeasy Mini Kit (Qiagen) and quantified with a NanoDrop ND 1000 spectrophotometer (NanoDrop Technologies, Wilmington, DE). Subsequently, 1 μg of total RNA was incubated with DNase (1U) and subjected to reverse transcription (RT) using the High-Capacity cDNA Reverse Transcription Kit (Thermo Fisher Scientific). Relative mRNA expression of genes associated with cell cycle regulation and apoptosis (Supporting Table 2) was quantified using Power SYBR Green Master Mix (Applied Biosystems) on an ABI Prism 7500 thermal cycler (Applied Biosystems). Expression values were normalized to β-actin, and relative abundance was calculated using ΔΔCt method.^48,49^

### Western Blotting

MCF-7 cells (1 × 10^6^ cells/100 mm plate) were treated with compounds **3** (10 and 20 μM) or **6** (3.5 and 7 μM) for 48 h. Proteins were extracted with RIPA buffer containing protease and phosphatase inhibitors (Sigma-Aldrich, #P8340) and quantified by BCA assay (Pierce). Samples (40 μg) were mixed with Laemmli buffer, denatured (100 °C, 5 min), separated on 12% SDS–PAGE (100 V, 2 h) and transferred (2 h, 200 mA) to PVDF membranes (Amersham Pharmacia). Membranes were blocked with 5% nonfat milk in TBS-T and incubated overnight at 4 °C with primary antibodies: Cyclin D1 (1:1000, Cell Signaling Technology, #55506) or Cyclin B1 (1:1000, CST, #12231), followed by HRP-conjugated secondary antibody (Invitrogen, #65–6120) for 2 h. β-actin (1:25000, Sigma-Aldrich, #A3854) was used as a loading control. Immunoreactive bands were detected using ECL Kit (Amersham Pharmacia) and quantified using ImageJ.^50^

### Statistical Analysis

Quantitative data are presented as mean ± standard deviation (SD) from at least three independent experiments. Significant differences among groups were evaluated by one-way ANOVA, followed by Tukey’s multiple comparison test. Analyses were performed using GraphPad Prism 8.0 (GraphPad Software, Inc.).

## Results

### Obtention of Sulfonylated Derivatives of Goniothalamin and Piplartine

Relying on theoretical predictions of their ADME properties obtained by established computational methods, we decided to synthesize the *O*-sulfonylated 2-pyridyl derivatives of goniothalamin (**1**) and piplartine (**4**). The process involved mixing pyridine-2-sulfonyl fluoride (PyFluor) and the corresponding phenolic analogue of the above natural products (**2** and **5**, respectively), in acetonitrile in the presence of cesium carbonate. A smooth reaction ensued which was completed after 1 h at room temperature, affording the desired derivatives **3** and **6**, respectively, in very high yields (93% and 92% yield, respectively) after purification by column chromatography.

The incorporation of the S=O group in derivatives **3** and **6** was evident from the strong, broad signal centered in 1379 and 1382 cm^–1^, respectively, assigned to the asymmetric stretching of the S=O group. Their complete structural characterization followed from the analysis of their ^1^H- and ^13^C NMR spectra and high-resolution mass spectrometry (HRMS) (Figures S1–S6). In the ^1^H NMR spectrum of goniothalamin derivative **3**, the pyridine signals were observed at 8.82 (d, *J* = 4.7 Hz, 1H), 8.02–7.88 (m, 2H), and 7.60 (ddd, *J* = 6.8, 4.7, 1.9 Hz, 1H), with the corresponding ^13^C signals at 153.6, 150.7, 149.5, 144.8, and 138.3 ppm. A similar pattern is present in the ^1^H NMR spectrum of piplartine derivative 6 with the hydrogen at carbon α to the pyridine nitrogen appearing at 8.79 (d, *J* = 4.7 Hz) in the ^1^H NMR spectrum and the remaining hydrogens of the pyridine ring in the range 8.07–7.53 ppm. The two carbons α to the nitrogen of pyridine ring were observed at 155.6 and 150.0 ppm in the ^13^C NMR spectrum. The exact masses of derivatives **3** and **6** were fully consistent with the expected molecular formulas of **3** (C_18_H_15_NO_5_S) and **6** (C_21_H_20_N_2_O_7_S).

### Compounds 3 and 6 Display Potent and Selective Antiproliferative Activity in MCF-7 Cells

Treatment of MCF-7 breast cancer cells with compounds **3** or **6** significantly reduced cell viability, with higher potency compared to their natural counterparts (compounds 1 and 4, respectively). Importantly, the selectivity of sulfonylated derivatives **3** and **6** toward tumor cells was markedly improved, being approximately 9-fold and 5-fold higher than that of compounds **1** and **4**, respectively. Classical chemotherapeutic agents doxorubicin and tamoxifen were used as positive controls, which displayed lower selectivity compared to compounds **3** and **6** (Figure 2A and Table 1). For subsequent investigations, the sulphonylated derivatives were used at concentrations corresponding to IC_50_ and IC_50_/2.

**Figure 2:**
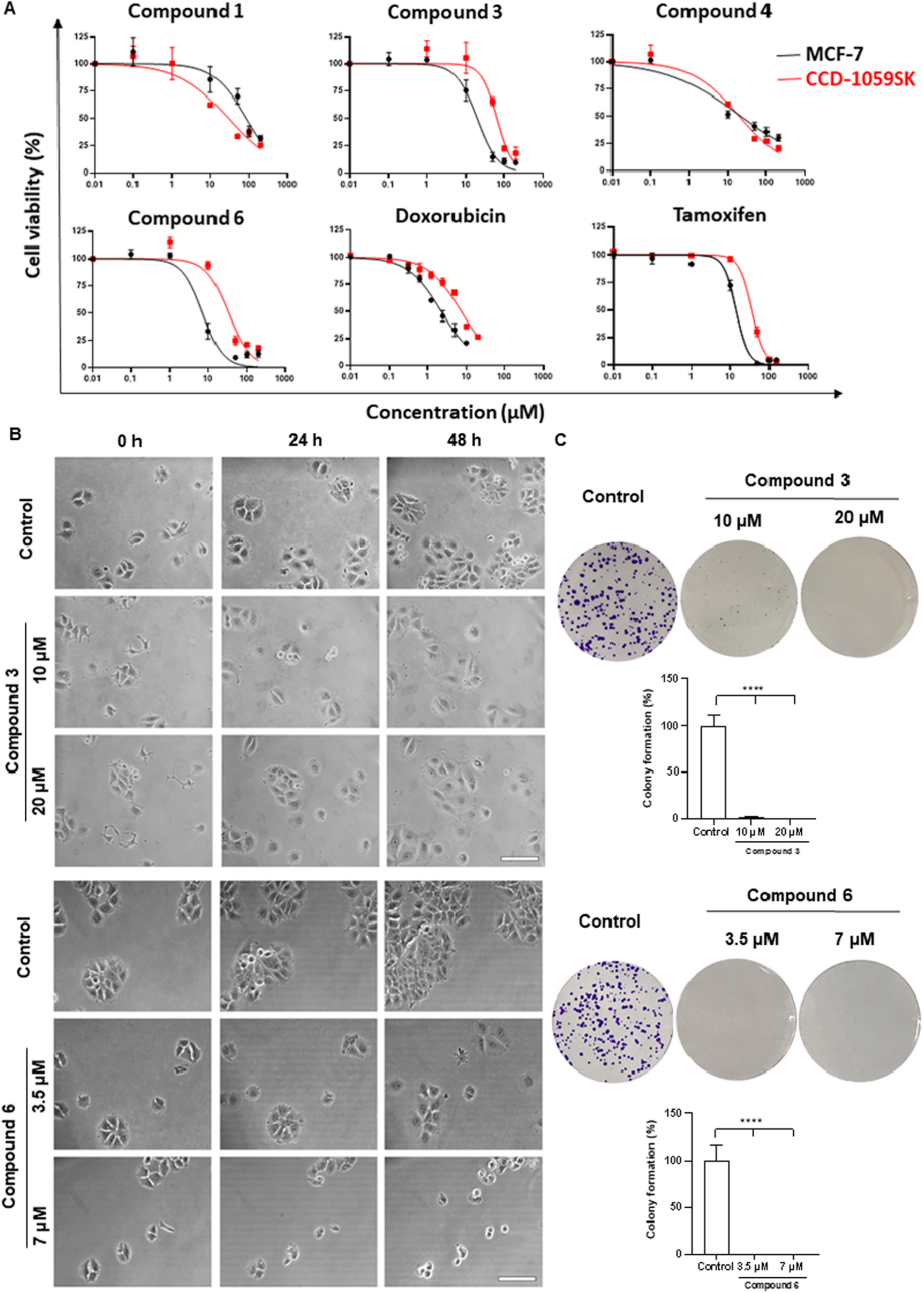
Compounds **3** and **6** show selective cytotoxicity against MCF-7 cells. (A) SRB dose–response curves after 48 h of treatment with compounds **1**, **3**, **4**, and **6** (0.01–200 μM), doxorubicin (0.01–20 μM) and tamoxifen (0.01–150 μM). (B) Phase-contrast images of MCF-7 cells treated with compounds **3** (10 and 20 μM) and **6** (3.5 and 7 μM) for 24 and 48 h.The images were captured from the same microscopic field throughout the culture period. Scale bar: 100 μm. (C) Clonogenic assay after 48 h treatment followed by 11-day recovery period. **** *p* < 0.0001 vs control ANOVA followed by Tukey’s post-test.

**Table 1:** IC_50_ Values (μM) Determined from Dose-Response Curves at 48 h of Treatment^a^

	MCF-7	CCD-1059SK	SI
compound 1	84.00 ± 1.04	29.01 ± 0.66	0.35
compound 3	19.69 ± 1.60	64.51 ± 2.15	3.28
compound 4	21.84 ± 0.45	19.82 ± 0.70	0.90
compound 6	7.12 ± 1.60	33.28 ± 4.49	4.67
doxorubicin	2.27 ± 0.10	7.39 ± 1.01	3.25
tamoxifen	14.17 ± 2.73	36.22 ± 2.62	2.55

a SI: Selectivity indices were determined as the ratio between IC_50_ values of nontumor (CCD-1059SK) and tumor (MCF-7) cells.

Morphological analysis revealed marked alterations in cell architecture following treatment with the tested substances. Compound **3** induced cell enlargement, particularly at 20 μM. Similarly, compound **6** promoted cell swelling at 3.5 μM, whereas at 7 μM, cells became rounded and detached, suggesting the onset of cell death (Figure 2B). Both compounds also impaired long-term proliferative capacity, as evidenced by a significant reduction in colony formation (Figure 2C).

To further explore the antiproliferative effects of the compounds **3** and **6**, we examined cell cycle progression. Compound **3** induced an accumulation of cells in G0/G1 and G2/M at 10 μM and 20 μM, respectively, compared to controls. An increased G2/M population was also observed in samples treated with compound **6** at 3.5 μM, whereas treatment at 7 μM resulted in a significant accumulation of sub-G1 population (dead cells) (Figures 3A, B and S7A, B).

**Figure 3:**
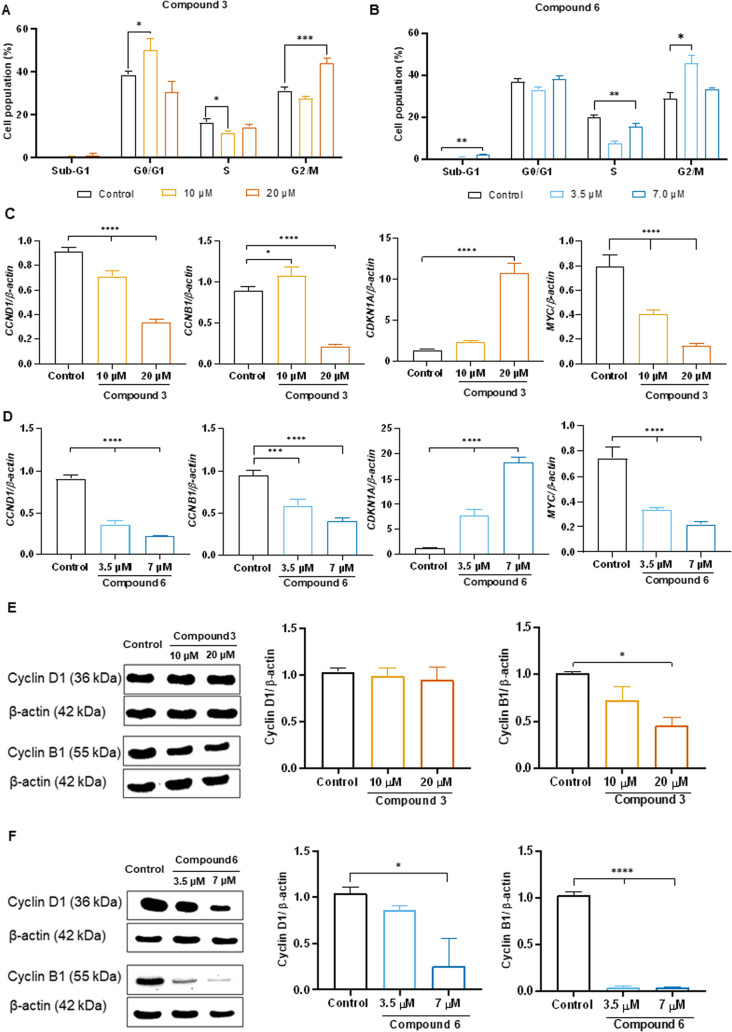
Compounds **3** and **6** modulate cell cycle progression and the expression of key regulators in MCF-7 cells. (A, B) Cell cycle distribution of samples treated for 48 h with compounds **3** (10 and 20 μM) or **6** (3.5 and 7 μM). (C, D) Relative mRNA expression determined after 24 h of treatment by qPCR of *CCNB1*, *CCND1*, *CDKN1A*, and *MYC*. (E, F) Protein levels of cyclins D1 and B1 after 48 h of treatment. β-actin was used as a loading control. *p < 0.05; **p < 0.01; ***p < 0.001; ****p < 0.0001 vs control, ANOVA followed by Tukey’s post-test.

The cell cycle arrest caused by compounds **3** and **6** was associated with their ability to modulate key regulatory genes. Compound **3** (10 and 20 μM) induced downregulation of *MYC* and *CCND1* transcripts, although cyclin D1 protein levels remained unchanged. Additionally, compound **3** at 20 μM reduced cyclin B expression (at both mRNA and protein levels) and promoted an increase in *CDKN1A* mRNA abundance (Figure 3C, E). Compound **6** also induced a downregulation of *CCND1* mRNA at both concentrations, with a significant reduction in cyclin D protein expression at 7 μM. Cyclin B and *MYC* expression were consistently decreased, while *CDKN1A* was upregulated at both 3.5 and 7 μM (Figure 3D, F).

### Compounds 3 and 6 Induced Cytoskeletal and Nuclear Alterations in MCF-7 Cells

The analysis of cytological preparations labeled for microtubules and actin filaments revealed pronounced morphological changes in MCF-7 cells exposed to compounds **3** and **6** (Figure 4 A, E). Treatment with compounds **3** (10 and 20 μM) or **6** (3.5 μM) resulted in increased cell size compared with controls, consistent with the alterations previously observed under phase-contrast microscopy. Compound **6** at 7 μM caused pronounced disruption of both the microtubule and microfilament networks (Figure 4E). A significant reduction in the number of cells per field was observed in cultures treated with both compounds (Figure 4B, F). Moreover, treated cells exhibited a reduced frequency of mitotic figures compared with untreated samples, which presented cells at various stages of division (Figure 4C, G). Nuclear abnormalities, including micronucleus formation, were also detected, supporting the genotoxic potential of these compounds (Figure 4D, H). Representative examples of the nuclear abnormalities considered for quantification are shown in Figure S8.

**Figure 4:**
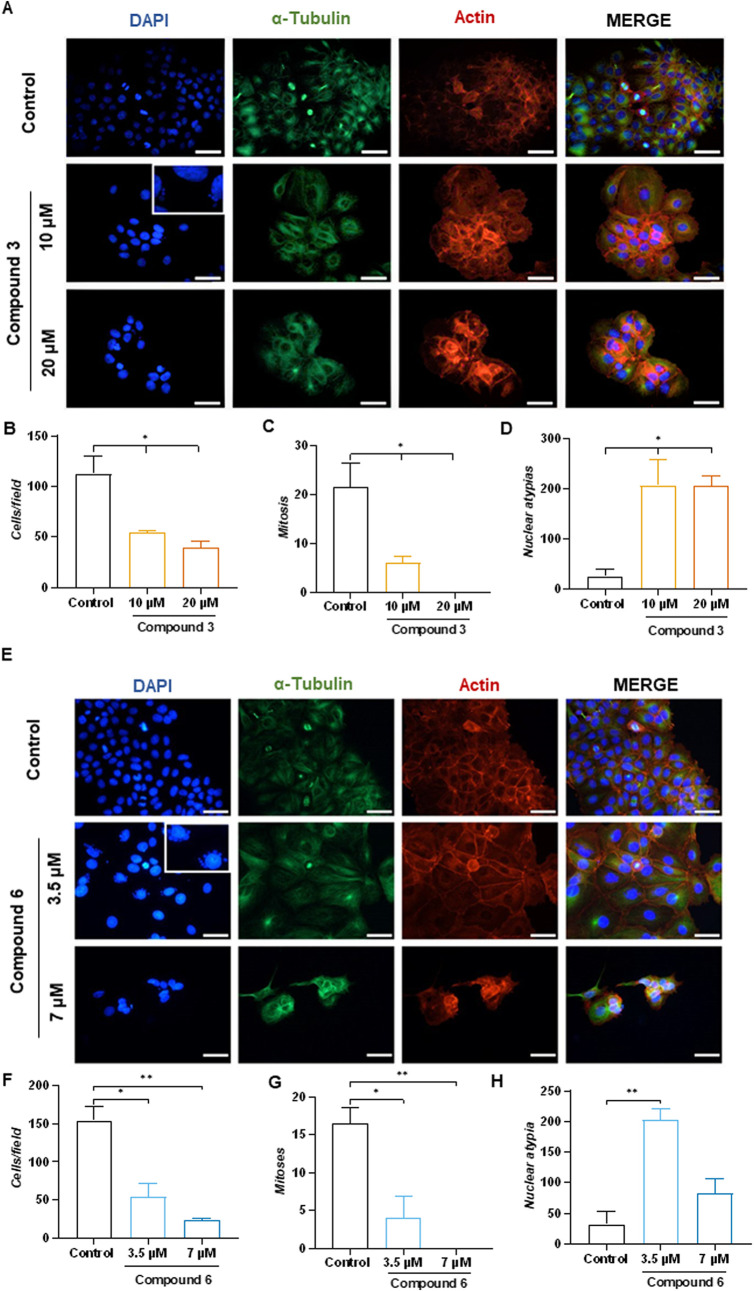
Compounds **3** and **6** induce cytoskeletal alterations and nuclear atypia in MCF-7 cells. (A, E) Representative fluorescence images of MCF-7 cells after 48 h of treatment with compounds **3** (10 and 20 μM) or **6** (3.5 and 7 μM), showing nuclear morphology (blue), α-tubulin (green), and actin filaments (red). Scale bar: 50 μm. (B, F) Quantification of cell number per field. (C, G) Frequency of mitotic cells. (D, H) Frequency of nuclear atypia, based on the evaluation of 1,000 cells per group. *p < 0.05, **p < 0.01 vs control, ANOVA followed by Tukey’s post-test.

### Compounds 3 and 6 Induced Oxidative Stress and DNA Damage in MCF-7 Cells

Given the nuclear atypia observed by immunocytochemistry, we hypothesized that compounds **3** and **6** could promote oxidative stress and DNA damage.^14,26^ Thus, we first assessed the protective effect of the antioxidant *N*-acetyl cysteine (NAC) on cell viability. Treatment with compounds **3** or **6** alone at IC_50_ concentration as expected markedly reduced cell viability compared with control (control = 100.0 ± 1.17%; **3** = 62.73 ± 6.27%; 6 = 56.30 ± 4.22%), while cotreatment with NAC significantly prevented this cytotoxic effect of these compounds (control = 99.22 ± 1.77%; **3** = 93.84 ± 3.64%; **6** = 93.21 ± 2.06%), indicating that oxidative stress is a central mechanism underlying the cytotoxicity activity of compounds **3** and **6** on MCF-7 cells (Figure 5A).

**Figure 5:**
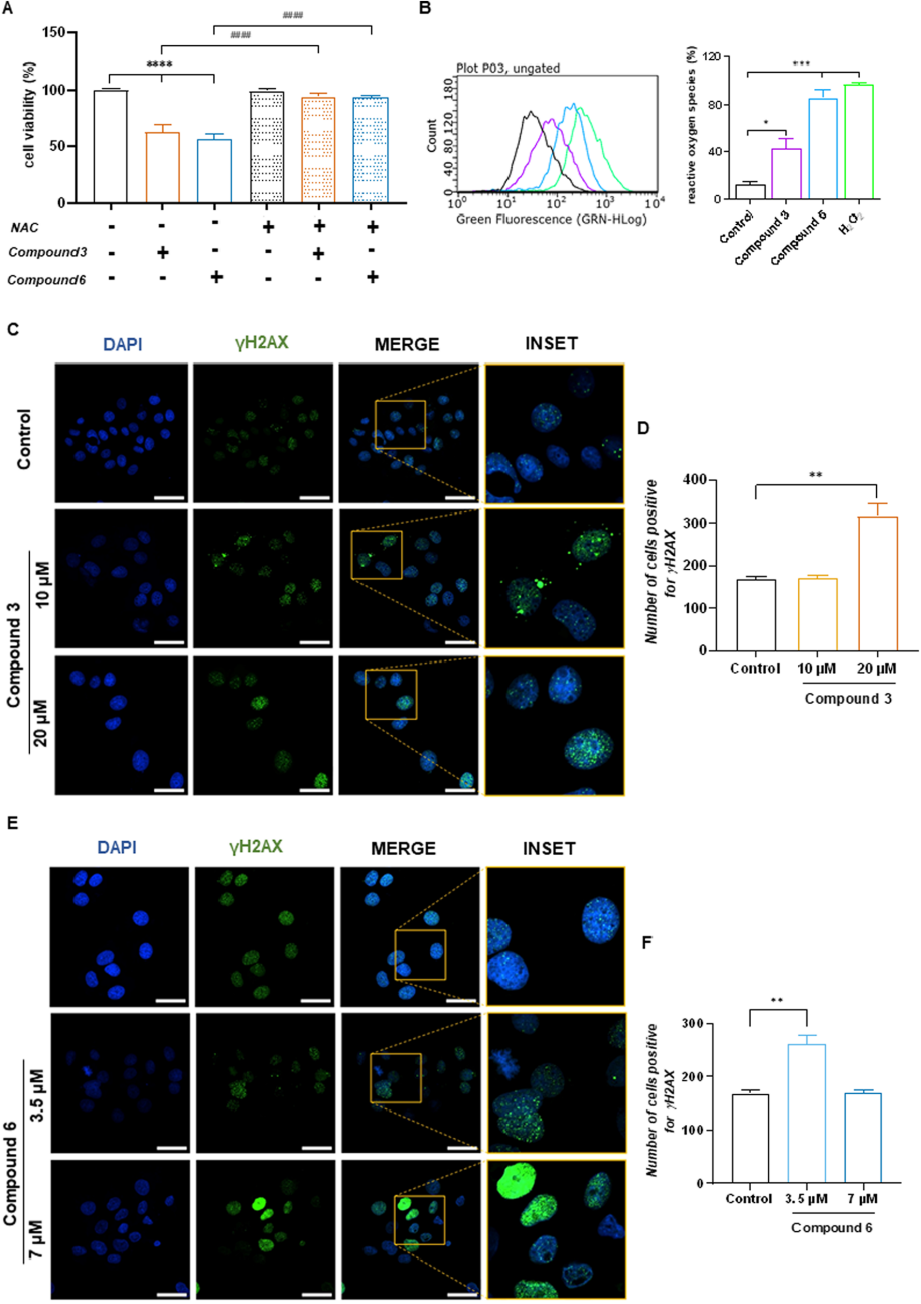
Compounds **3** and **6** induce oxidative stress and DNA damage in MCF-7 cells. (A) Cell viability after 48 h of treatment with compounds **3** (20 μM) or **6** (7 μM) in presence or absence of NAC (1 mM). (B) Representative histograms and quantitative analysis of intracellular ROS (%) after 4 h of treatment with compound **3** (20 μM) or **6** (7 μM). H_2_O_2_ (1 mM, 30 min) was used as a positive control. (C, E) Representative confocal images of nuclei (blue) and γH2AX (green) following 24 h of treatment with compounds **3** (10 and 20 μM) or **6** (3.5 and 7 μM). Scale bar: 50 μm. (D, F) Quantification of γH2AX positive cells after 24 h of treatment. #### *p* < 0.0001 when comparing treated groups in the presence or absence of NAC. *p < 0.05, **p < 0.01, ***p < 0.001, ****p < 0.0001 vs control, ANOVA followed by Tukey’s post-test.

Consistently, the CellROX assay revealed a strong pro-oxidant effect of tested compounds. After 4 h of exposure, compound **3** increased ROS production to 42.46 ± 8.99% compared with control (12.46 ± 2.1%), whereas compound **6** caused an even stronger effect (85.78 ± 6.6%), approaching ROS levels induced by positive control H_2_O_2_ (96.74 ± 1.46%) (Figures 5B and S9).

We next investigated whether this oxidative stress translated into DNA damage. Immunodetection of phosphorylated histone H2A.X (γH2AX), a key marker of the DNA damage response (DDR), revealed an increased frequency of positive cells for this marker after 24 h of treatment with compound **3** (20 μM) or compound **6** (3.5 μM). Although the samples treated with compound **6** at 7 μM did not show a significant difference in the frequency of cells positive for γH2AX compared to the control groups, intense nuclear staining was observed, indicating substantial DNA damage (Figure 5C, D, E, F). Taken together, these results demonstrate that compounds **3** and **6** exert their cytotoxic effects on MCF-7 cells, at least in part, through the induction of oxidative stress and consequent DNA damage.

### Compound 6 Induced Apoptosis, While Compound 3 Induced Senescence in MCF-7 Cells

To investigate the response of MCF-7 cells to oxidative stress and DNA damage, we performed the annexin V/propidium iodide (PI) double-staining assay and RT-qPCR for BAX and BCL2 to assess apoptosis induction. Treatment with compound **3** did not significantly increase the proportion of apoptotic cells compared with controls (10 μM = 9.14% ± 3.0%, 20 μM = 9.81% ± 1.28% and control = 7.19% ± 0.72%) (Figure 6A, B). Similarly, no changes were observed regarding positive cells for annexin V in samples treated with compound **6** at 3.5 μM compared to the control group (treated = 8.4% ± 0.25% and control = 5.95% ± 1.36%). In contrast, compound **6** at 7 μM markedly increased the frequency Annexin V-positive cells (46.32% ± 3.86%), consistent with increased sub-G1 population previously observed in cell cycle analysis (Figure 6C, D). Both compounds also modulated the expression of apoptosis-related genes. After 24 h of treatment, compounds **3** (10 and 20 μM) and **6** (3.5 and 7 μM) induced upregulation of the pro-apoptotic *BAX* gene (Figure 6E, G) and downregulation of the antiapoptotic *BCL2* gene (Figure 6F, H).

**Figure 6:**
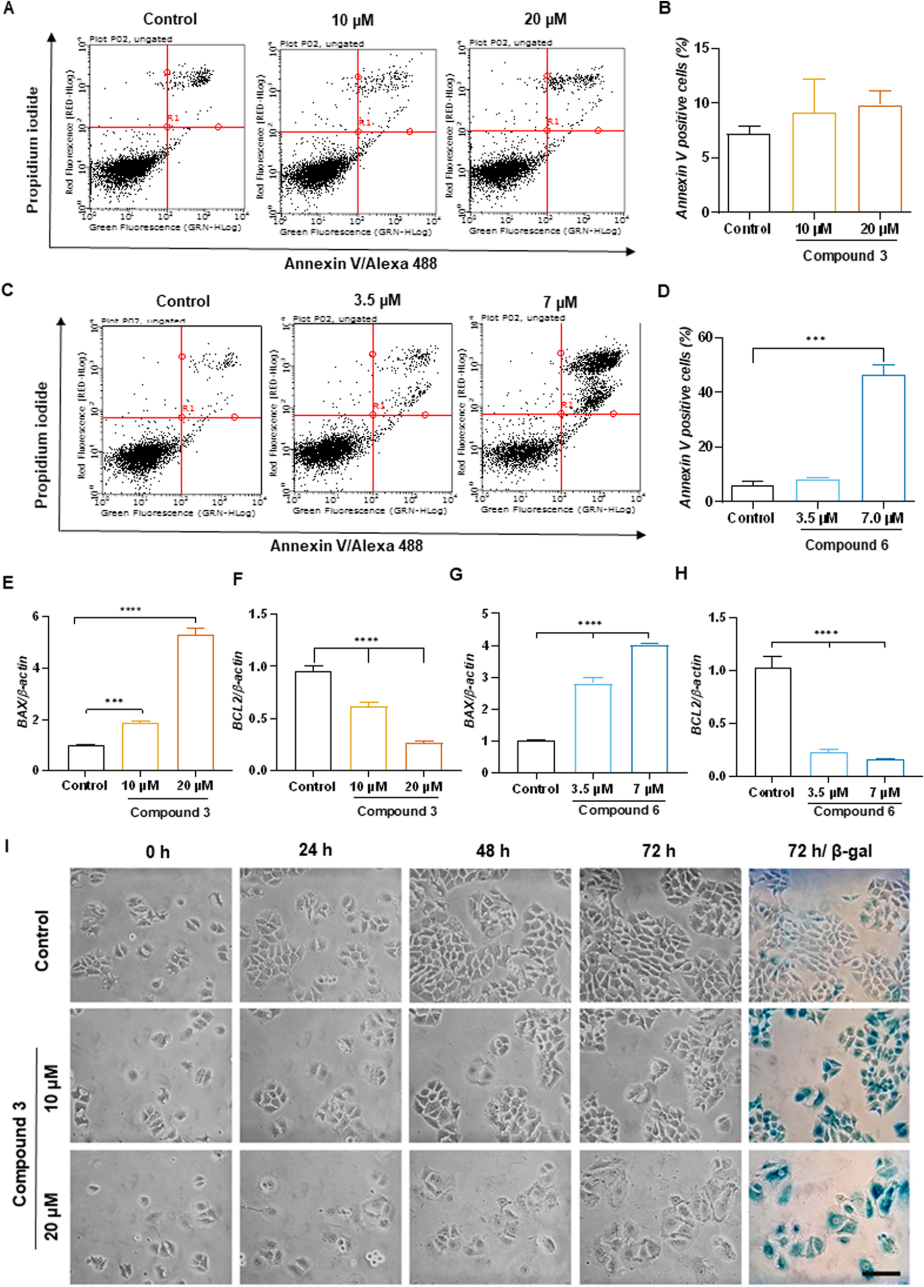
Compound **6** induces apoptosis, while compound **3** promotes senescence in MCF-7 cells. (A, C) Representative Annexin V/PI dot plots after 48 h of treatment with compounds **3** (10 and 20 μM) or **6** (3.5 and 7 μM), showing viable cells (lower left), early apoptosis (lower right), late apoptosis (upper right), and necrotic cells (upper left). (B, D) Quantification of Annexin V-positive cells after 48 h. (E, G) Relative *BAX* mRNA expression after 24 h. (F, H) Relative *BCL2* mRNA expression after 24 h. (I) Representative phase-contrast images showing the same microscopic field at 24, 48, and 72 h after treatment with compound **3**. β-Galactosidase staining was performed after 72 h of treatment. Scale bar: 100 μm. ***p < 0.001, ****p < 0.0001 vs control, ANOVA followed by Tukey’s post-test.

Since compound **3** did not induce apoptosis despite altering *BAX* and *BCL2* expression, we further evaluated its effect on the cellular senescence process. Treatment with compound **3** inhibited proliferation and caused pronounced morphological changes, including cell enlargement. These alterations were accompanied by increased β-galactosidase staining after 72 h at both 10 and 20 μM, indicating that the treatment was effective in inducing senescence in MCF-7 cells (Figure 6I).

## Discussion

In this study, we investigated the antitumor potential of sulfonylated derivatives of goniothalamin (**1**) and piplartine (**4**), namely compounds **3** and **6**, respectively, using estrogen receptor-positive breast cancer cells (MCF-7) as a study model. Both derivatives exhibited enhanced potency to reduce cell viability with notable improvements of selectivity toward tumor cells compared with their parental natural products. Compounds **3** and **6** were 9-fold and 5-fold more selective than **1** and **4**, respectively. Despite the limited number of examples which precludes (SAR) studies, the findings suggest that the sulfonyl group contributed significantly to the improved cytotoxic activity and selectivity profiles, consistent with observations reported by Bernal and co-workers.^51^ Primary dermal fibroblasts (CCD-1059Sk) were used as control of normal cells to evaluate selectivity of the tested compounds considering that primary cultures retain with more fidelity the in vivo cellular behavior.

The data from clonogenic assay revealed that compounds **3** and **6** effectively suppress the long-term proliferative capacity of MCF-7 cells, reinforcing that sulfonylation represents a valuable chemical strategy for optimizing the pharmacological properties of GNT (**1**) and PPT (**4**). Further, mechanisms underlying cytotoxic and antiproliferative effects of these compounds on MCF-7 cells were explored. The compounds **3** and **6** disrupted redox balance in MCF-7 by increasing intracellular ROS levels, which promoted DNA damage and, ultimately, cellular senescence or cell death. These findings indicate that the structural modifications preserved redox-modulating activity previously observed for compounds **1** and **4**,^52–56^ while enhancing their antitumor effects.

DNA damage caused in MCF-7 cells in response to the treatments was demonstrated by significant increase in the frequency of γH2AX-positive cells and nuclear abnormalities, including micronucleation and multinucleation. γH2AX is a well-established marker of DNA double-strand breaks, which is generated by ATM, ATR, and DNA-PK kinases and contributes to the recruitment of DNA damage response (DDR) and repair proteins.^57–59^ Importantly, our data revealed that the magnitude of DNA damage induced by compounds **3** and **6** exceeds the repair capacity of MCF-7 cells, resulting in irreversible DNA damage, cell cycle arrest and, subsequently, apoptosis or senescence.

Compounds **3** and **6** induced accumulation of cells in the G0/G1 and G2/M phases, which was accompanied by downregulation of *MYC*, *CCND1* (cyclin D1), and *CCNB1* (cyclin B1), oncogenes commonly overexpressed in breast cancer and associated with tumor progression and chemoresistance.^60–65^ Concomitantly, there was upregulation of *CDKN1A* (p21), a master inhibitor of cyclin-CDK complexes, whose transcription is primarily controlled by the tumor suppressor p53. Given that the MCF-7 cell line is wild-type for the *TP53* gene (p53), it is plausible that activation of the p53–p21 axis in response to ROS-mediated DNA damage has contributed to cell cycle arrest or cell death^66–69^ observed in MCF-7 in response to the treatments.

Additionally, compound **6** at higher concentration clearly altered microtubule organization; however, its direct interaction with tubulin was not assessed in this study. Piplartine (**4**), the natural parent of compound **6**, contains a trimethoxyphenyl moiety that has been associated with binding to the colchicine site on tubulin, similarly to other microtubule-targeting agents such as combretastatin A-4 and chalcone derivatives.^70,71^ Although compound **6** does not retain exact trimethoxy substitution pattern, it preserves a methoxy-substituted aromatic group, which may contribute to interactions with tubulin or related targets, although this remains speculative. Importantly, immunofluorescence analysis revealed a clear disruption of microtubule organization in response to treatment with compound **6**, indicating that this compound affects cytoskeleton dynamics. Whether this effect results from direct interaction with tubulin or occurs indirectly remains to be determined. Notably, these alterations occur in parallel with ROS-mediated DNA damage, supporting a multifactorial mechanism of action involving both cytoskeletal disruption and genotoxic stress. Further studies will be required to clarify the relative contribution of these mechanisms.

We also sought to investigate cell fate in response to oxidative stress and genotoxic effects induced by the treatments. The data revealed that compounds **3** and **6** lead to distinct cell fates. While compound **3** predominantly promoted senescence, as evidenced by increased β-galactosidase activity and morphological changes, compound **6** induced apoptosis, as indicated by an increased proportion of Annexin V–positive cells and modulation of the expression of the pro-apoptotic gene BAX (upregulation) and the antiapoptotic gene BCL2 (downregulation).

Cellular senescence induction is recognized as a tumor-suppressive mechanism depending on context, and the potential contribution of the senescence-associated secretory phenotype (SASP) must be further clarified, given its dual role in either reinforcing tumor suppression or promoting tumor progression.^72–77^ Already, the ability of compound **6** to induce apoptosis in MCF-7 cells highlights its translational potential as a cytotoxic agent capable of overcoming resistance mechanisms associated with defective apoptotic signaling.^78–80^ Future studies, particularly in vivo investigations, will be essential to define pharmacokinetic properties of the studied compounds, validate their antitumor activity, and evaluate the therapeutic relevance of distinct mechanisms of tumor suppression.

## Conclusion

Taken together, this study demonstrates the antitumor potential of sulfonylated derivatives of GNT (compound **3**) and PPT (compound **6**), which exhibit enhanced potency and selectivity toward MCF-7 breast cancer cells compared to their natural counterparts. Notably, these compounds trigger distinct tumor-suppressive programs, with compound **3** inducing senescence and compound **6** promoting apoptosis, both in association with ROS-mediated DNA damage. These findings highlight sulfonylation as an effective chemical strategy to improve the biological activity of natural scaffolds while modulating their mechanisms of action. Collectively, this work provides a foundation for the rational design of novel anticancer agents targeting breast cancer.

## Supplementary Material



## Notes

Some calculated physicochemical parameters for the compounds (Table S1); summarizes the primer sequences used for RT-qPCR analysis (Table S2); provide the chemical characterization of compounds **3** and **6** through ^1^H NMR, ^13^C NMR, and HRMS spectra (Figures S1–S6); shows representative histograms used in cell cycle analysis (Figure S7); exhibits representative immunofluorescence images to illustrate the cytoskeletal distribution pattern and nuclear alterations (Figure S8); shows representative dot plots from flow cytometry analysis of intracellular reactive oxygen species quantification (Figure S9) (PDF)

## §

Laboratório de Neuroproteômica (LNP), Instituto de Biologia, Universidade Estadual de Campinas (UNICAMP), Campinas, SP, zip code 13083–862, Brazil
