# Supplementary material for: Sulfonylated Goniothalamin and Piplartine Derivatives Exhibit Selective Antiproliferative Activity on Breast Cancer Cells via Oxidative Stress-Mediated Mechanisms
Source: ACS Omega. 2026 Jul 7;11(28):41442–55. doi: 10.1021/acsomega.6c00212 (PMC13393042; doi:10.1021/acsomega.6c00212)
Supplement: Supplementary file 1 [file ao6c00212_si_001.pdf]

Sulfonylated goniothalamin and piplartine derivatives exhibit selective antiproliferative activity on breast cancer cells via oxidative stress-mediated mechanisms

Julia Louise Moreira Nacif[1]†, Aloisio de Andrade Bartolomeu[2], Simone da Silva Lamartine-Hanemann[1], Bruno Zavan[1], Luiza de Mello Nascimento[1], Ester Siqueira Caixeta[1], Alexandre Ferro Aissa[1], Ronaldo Aloise Pilli[2], Marisa Ionta[1]

1. Laboratório de Avaliação de Protótipos Antitumorais (LAPAN), Instituto de Ciências Biomédicas, Universidade Federal de Alfenas, Alfenas, Minas Gerais, zip code 37-130-001, Brazil.
2. Instituto de Química, Universidade Estadual de Campinas (UNICAMP), Campinas, SP, zip code 13083-970, Brazil.

† Current address: Laboratório de Neuroproteômica (LNP), Institudo de Biologia, Universidade Estadual de Campinas (UNICAMP), Campinas, SP, zip code 13083-862, Brazil

Supporting Information

Table S1. Physicochemical parameters determined for assayed compounds.

Compounds	**cLogP**	**Heavy atoms**	**TPSA**	**HBA**	**MW**	**LE**	**LLE**	**SEI**	**BEI**
**1**	2.61	15	26.30	2	200.23	0.380	1,47	15.50	20.35
**3**	2.81	25	90.00	6	357.38	0.261	1.85	5.17	13.04
**4**	1.96	23	65.07	5	317.34	0.286	2.75	7.23	14.83
**6**	2.19	31	120.48	8	444.46	0.232	2.96	4.27	11.58

cLogP: calculated logarithm of the partition coefficient; heavy atoms: any atom except hydrogen; TPSA: topological surface area; HBA: hydrogen bond acceptor; MW: molecular weight ($g.mol^{-1}$); LE: ligand efficiency; LLE: lipophilic ligand efficiency; SEI: surface efficiency index: BEI: binding efficiency index.

Table S2. Primer sequences used for RT-qPCR analysis.
List of forward (F) and reverse (R) primer sequences designed for amplification of target genes associated with cell cycle regulation and apoptosis (*CDKN1A, CCNB1, CCND1, BAX, BCL2, MYC*). β-actin (ACTB) was used as the endogenous control for normalization.

Gene	Primer sequence (5’ → 3’)
CDKN1A	F: CCATAGCCTCTACTGCCACCATC<br>R: GTCCAGCGACCTTCCTCATCCA
CCNB1	F: GTACCCTCCAGAAATTGGTGA<br>R: GACTACATTCTTAGCCAGGTG
CCND1	F: GGGTTGTGCTACAGATGATAGAG<br>R: AGACGCCTCCTTTGTGTTAAT

BAX	F: TTCCTTACGTGTCTGATCAATCC<br>R: GGGCAGAAGGCACTAATCAA
BCL2	F: CAGAAGTCTGGGAATCGATCTG<br>R: AATCTTCAGCACTCTCCAGTTATAG
MYC	F: AAGCTGAGGCACACAAAGA<br>R: GCTTGGACAGGTTAGGAGTAAA
ACTB	F: AGAGCTACGAGCTGCCTGAC<br>R: AGCACTGTGTTGGCGTACAG

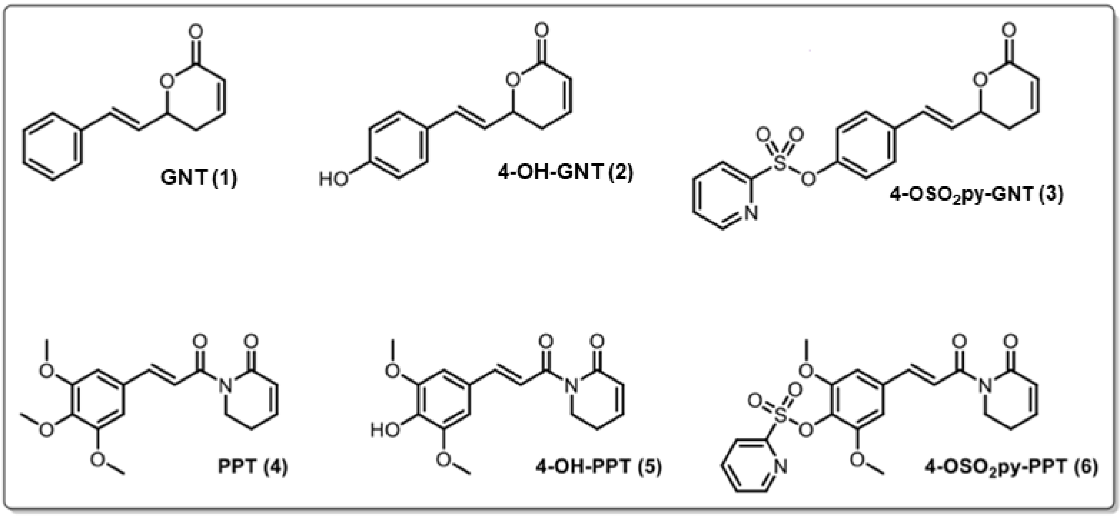

Figure S1. Chemical structures of natural products goniothalamin (GNT, **1**) and piplartine (PPT, **4**) and their hydroxylated derivatives 4-OH-GNT (**2**) and 4-OH-PPT (**5**) and sulfonylated derivatives 4-(2'-SO2pyr)O-GNT (**3**) and 4-(2'-SO2pyr)O-PPT (**6**).

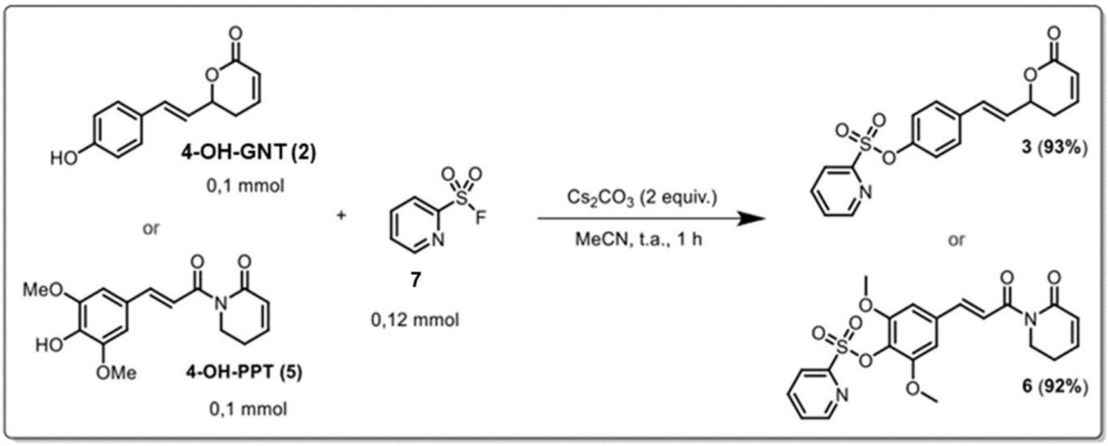

Figure S2. Synthetic routes for obtaining sulfonylated derivatives of GNT and PPT. Reaction of 4-OH-GNT (**2**) or 4-OH-PPT (**5**) with pyridine-3-sulfonyl fluoride (**7**) in the presence of Cs_2CO_3 afforded the corresponding aryl sulfonates (**3**) and (**6**) in 93% and 92% yields, respectively.

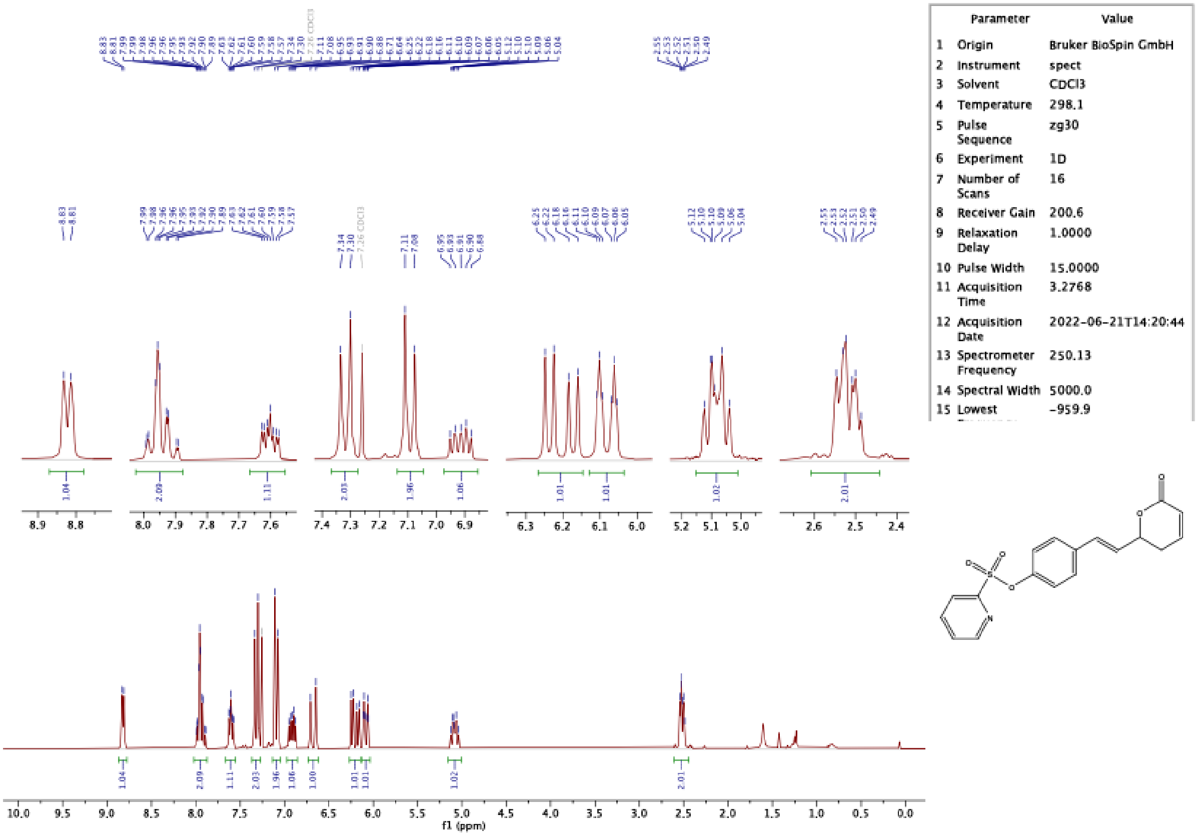

Figure S3.^{1}H NMR spectrum of *rac*-(*E*)-4-(2-(6-oxo-3,6-dihydro-2*H*-pyran-2-yl)vinyl)phenyl pyridine-2-sulfonate (**3**).

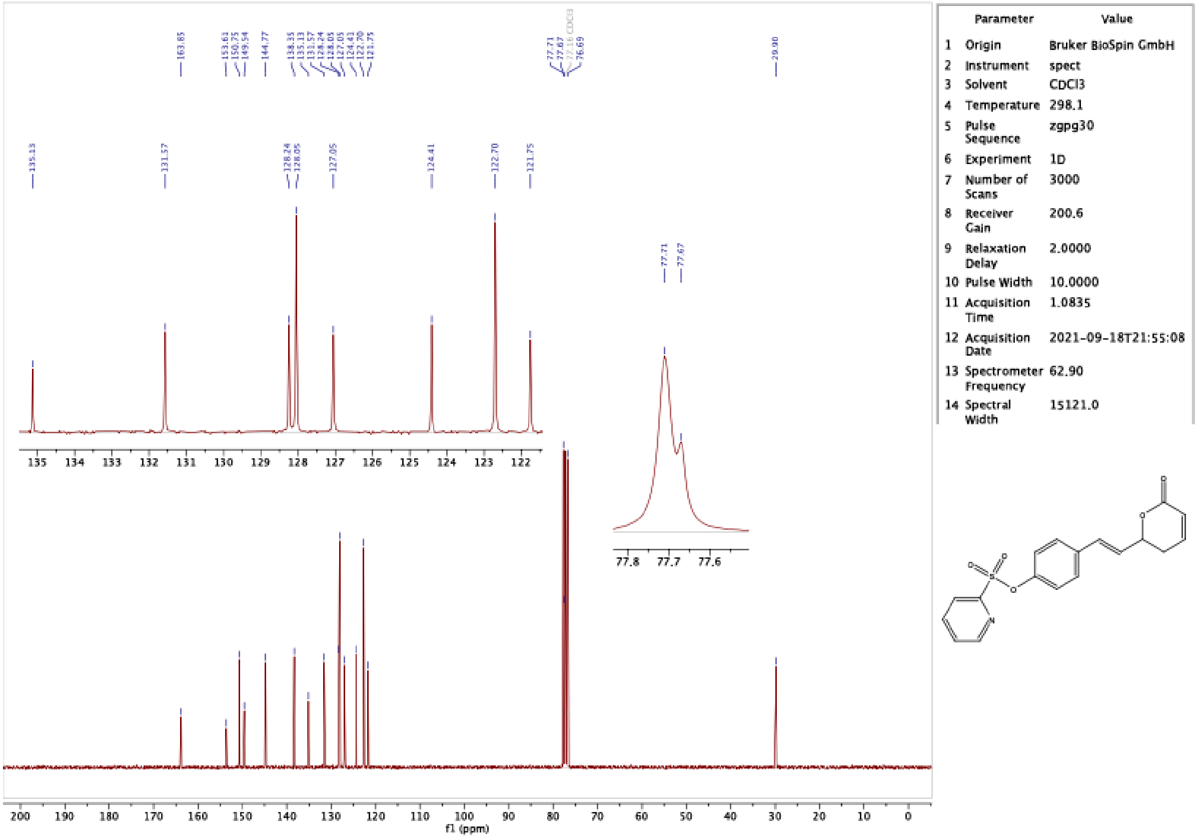

	Parameter	Value
1	Origin	Bruker BioSpin GmbH
2	Instrument	spect
3	Solvent	CDCl3
4	Temperature	298.1
5	Pulse Sequence	zgpg30
6	Experiment	1D
7	Number of Scans	3000
8	Receiver Gain	200.6
9	Relaxation Delay	2.0000
10	Pulse Width	10.0000
11	Acquisition Time	1.0835
12	Acquisition Date	2021-09-18T21:55:08
13	Spectrometer Frequency	62.90
14	Spectral Width	15121.0

Figure S4. ^{13}C NMR spectrum of *rac*-(*E*)-4-(2-(6-oxo-3,6-dihydro-2*H*-pyran-2-yl)vinyl)phenyl pyridine-2-sulfonate (**3**).

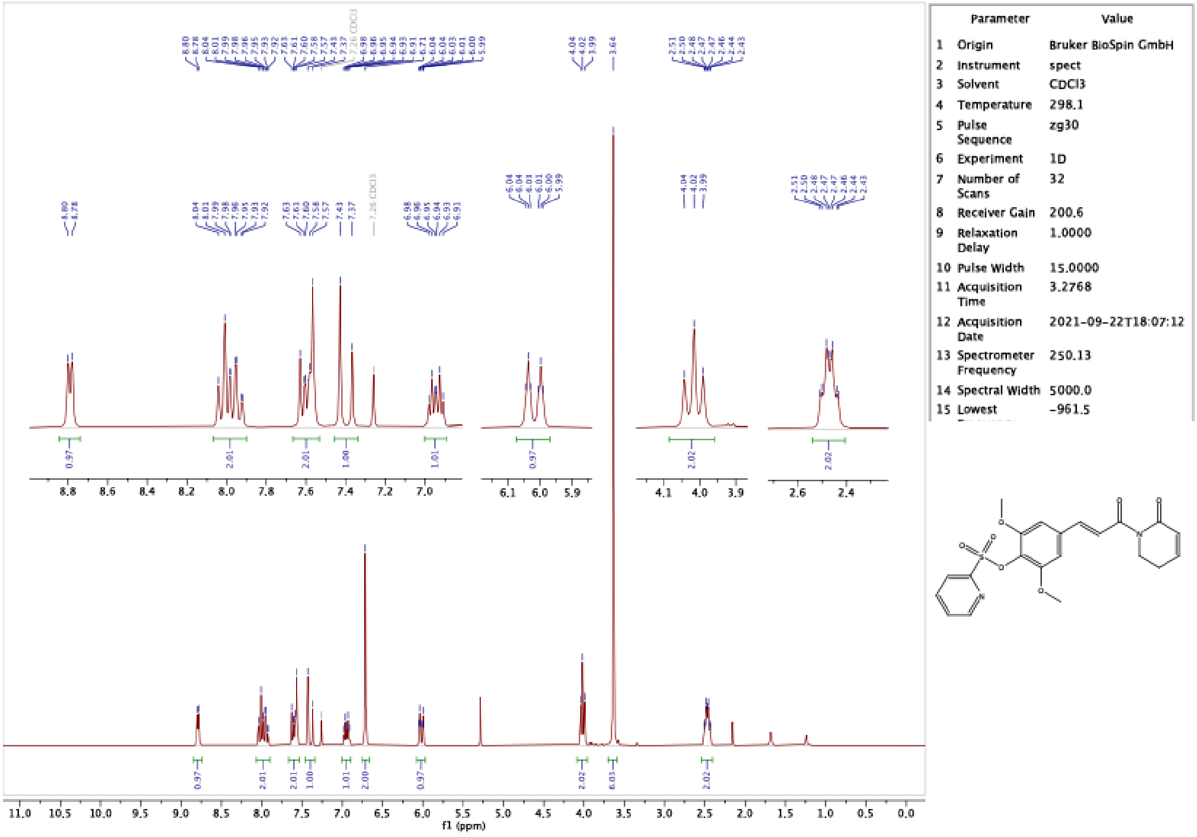

Figure S5. ^{1}H NMR spectrum of (*E*)-2,6-dimethoxy-4-(3-oxo-3-(6-oxo-3,6-dihydropyridin-1(2*H*)-yl)prop-1-en-1-yl)phenyl pyridine-2-sulfonate (**6**).

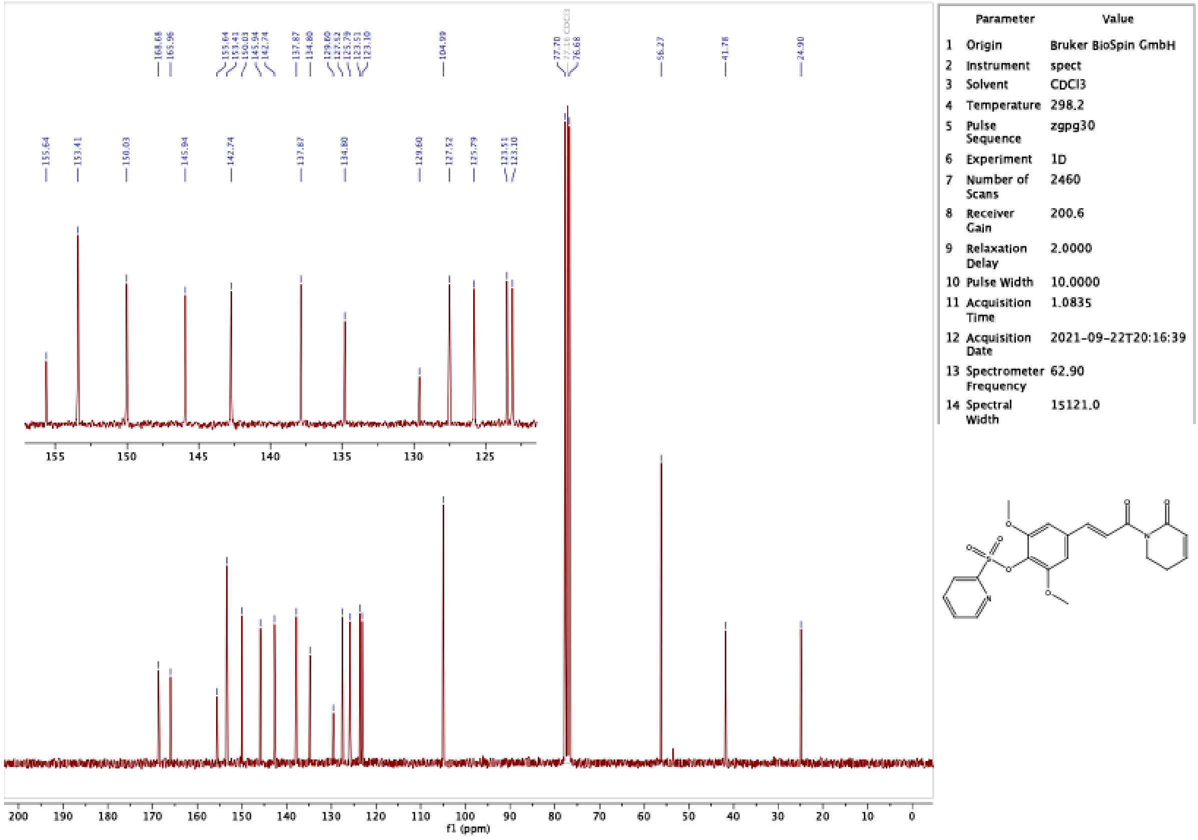

Figure S6. ^{13}C NMR spectrum of (*E*)-2,6-dimethoxy-4-(3-oxo-3-(6-oxo-3,6-dihydropyridin-1(2*H*)-yl)prop-1-en-1-yl)phenyl pyridine-2-sulfonate (**6**).

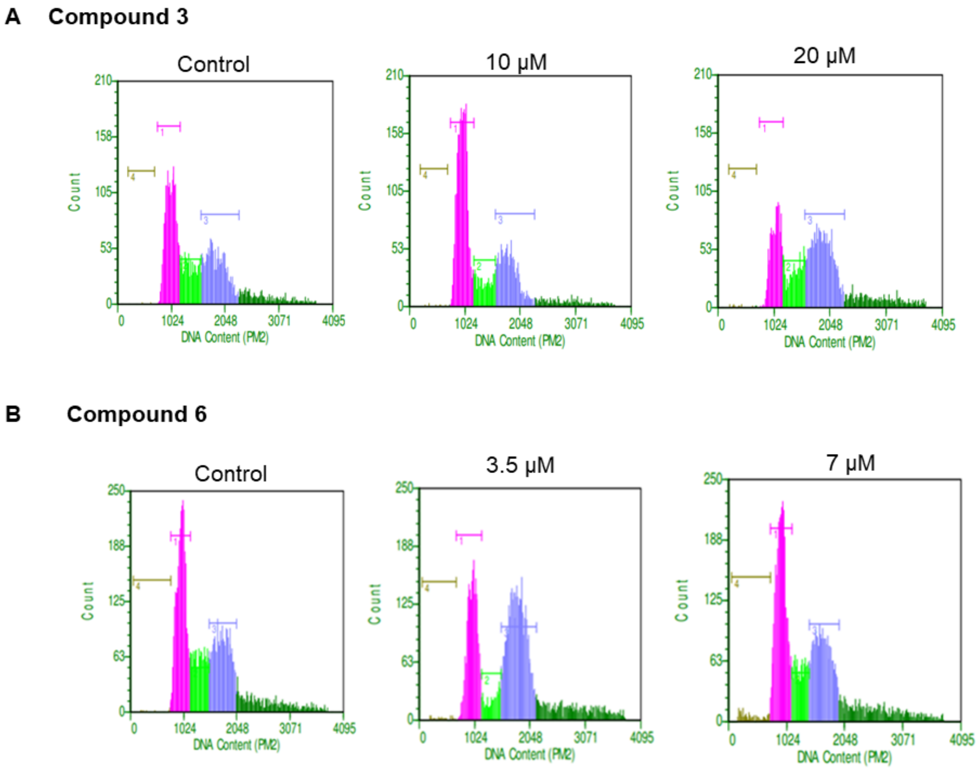

Figure S7. (A) Representative histograms of the cell cycle assay in MCF-7 cells after 48 h of treatment with compound **3** (10 and 20 µM). Representative histograms of the cell cycle assay in MCF-7 cells after 48 h of treatment with compound **6** (3.5 and 7 µM).

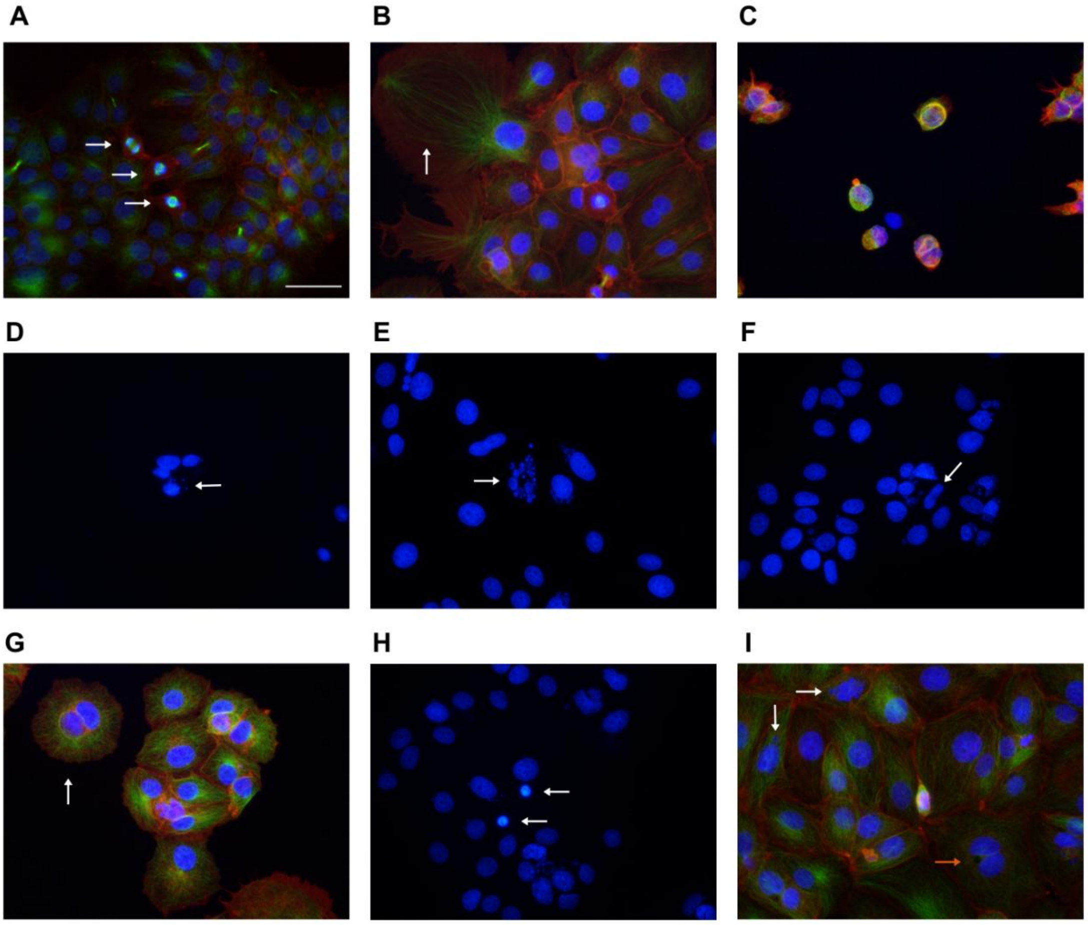

Figure S8. Representative immunofluorescence images showing cytoskeletal and nuclear alterations: nuclei (blue), α-tubulin (green), and actin filaments (red). (A) Mitotic cell (white arrows, control). (B–C) Cytoskeletal alterations (white arrows). (D) Micronuclei (white arrows). (E) Nuclear fragmentation (white arrows). (F) Irregular nuclear surface (white arrows). (G) Binucleation (white arrows). (H) Pyknotic nucleus (white arrows). (I) Multinucleation (white arrows) and binucleation (orange arrow). Images B–I were obtained from cells treated with compounds **3** or **6**. Scale bar: 50 µm.

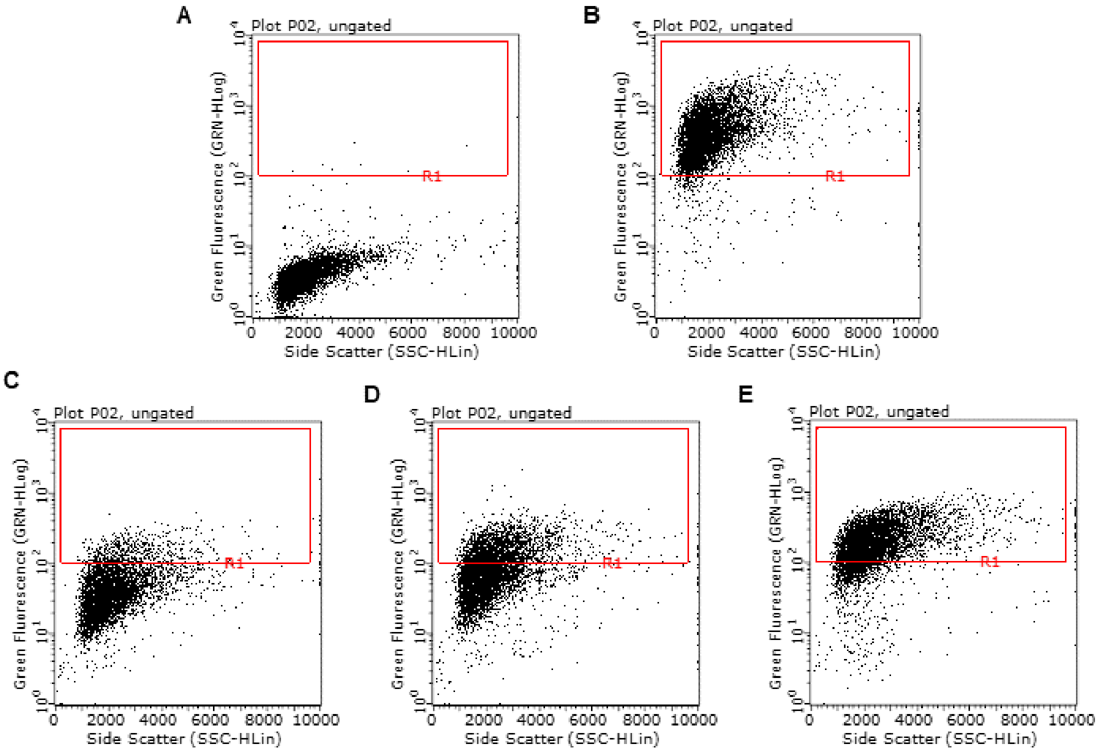

Figure S9. Representative dot plots of reactive oxygen species (ROS) production. (A) Unstained cells, used as a negative control to define the positive region R1. (B) Cells treated with hydrogen peroxide (H_2O_2; 1 mM; 30 min), used as a positive control. (C) Control group cells (DMSO). (D) Cells treated with compound **3** (20 µM; 4 h). (E) Cells treated with compound **6** (7 µM; 4 h).
